# Process evaluation of the *Getting it Right* study and acceptability and feasibility of screening for depression with the aPHQ-9

**DOI:** 10.1186/s12889-019-7569-4

**Published:** 2019-09-18

**Authors:** Sara Farnbach, Graham Gee, Anne-Marie Eades, John Robert Evans, Jamie Fernando, Belinda Hammond, Matty Simms, Karrina DeMasi, Nick Glozier, Alex Brown, Maree L. Hackett, on behalf of the Getting it Right Investigators, Maree Hackett, Armando Teixeira-Pinto, Nick Glozier, Timothy Skinner, Deborah Askew, Graham Gee, Alan Cass, Alex Brown

**Affiliations:** 1 The George Institute for Global Health, The University of New South Wales, PO Box M201, Missenden Road, Sydney, New South Wales 2050 Australia; 2 University of New South Wales, Sydney, New South Wales 2052 Australia; 3 University of Sydney, Sydney, New South Wales 2006 Australia; 4 Victorian Aboriginal Health Service, Melbourne, Victoria 3065 Australia; 5 University of Melbourne, Melbourne, Victoria 3000 Australia; 6 Murdoch Children’s Research Institute, Melbourne, Victoria 3052 Australia; 7 The University of Technology, Sydney, New South Wales 2006 Australia; 8 The University of Sydney, Sydney, New South Wales 2006 Australia; 9 The Glen Centre (Ngampie), Chittaway Point, New South Wales 2261 Australia; 10 The University of Newcastle, Newcastle, New South Wales 2308 Australia; 11 Nunkuwarrin Yunti of South Australia, Adelaide, South Australia 5000 Australia; 12 Aboriginal Medical Services Alliance Northern Territory, Darwin, 0801 Australia; 13 Brain and Mind Centre and Central Clinical School University of Sydney, Sydney, New South Wales 2052 Australia; 14 Wardliparingga Aboriginal Research Unit, South Australian Health and Medical Research Institute, Adelaide, SA Australia; 15 Sansom Institute for Health Research, University of South Australia, Adelaide, SA Australia; 16 The University of Central Lancashire, Preston, PR1 2HE UK

**Keywords:** Indigenous health, Social and emotional wellbeing, Depression screening, Primary healthcare, Process evaluation, Validation study

## Abstract

**Background:**

The Getting it Right study determined the validity, sensitivity, specificity and acceptability of the culturally adapted 9-item Patient Health Questionnaire (aPHQ-9) as a screening tool for depression in Aboriginal and Torres Strait Islander (hereafter referred to as Indigenous) people. In this process evaluation we aimed to explore staff perceptions about whether Getting it Right was conducted per protocol, and if the aPHQ-9 was considered an acceptable and feasible screening tool for depression in primary healthcare. This process evaluation will provide information for clinicians and policy makers about the experiences of staff and patients with Getting it Right and what they thought about using the aPHQ-9.

**Methods:**

Process evaluation using grounded theory approaches. Semi-structured interviews with primary healthcare staff from services participating in Getting it Right were triangulated with feedback (free-text and elicited) from participants collected during the validation study and field notes. Data were thematically analysed according to the Getting it Right study protocol to identify the acceptability and feasibility of the aPHQ-9.

**Results:**

Primary healthcare staff (*n* = 36) and community members (*n* = 4) from nine of the ten participating Getting it Right services and Indigenous participants (*n* = 500) from the ten services that took part. Most staff reported that the research was conducted according to the study protocol. Staff from two services reported sometimes recruiting opportunistically (rather than recruiting consecutive patients attending the service as outlined in the main study protocol), when they spoke to patients who they knew from previous interactions, because they perceived their previous relationship may increase the likelihood of patients participating. All Getting it Right participants responded to at least six of the seven feedback questions and 20% provided free-text feedback. Most staff said they would use the aPHQ-9 and most participants said that the questions were easy to understand (87%), the response categories made sense (89%) and that they felt comfortable answering the questions (91%).

**Conclusion:**

Getting it Right was predominantly conducted according to the study protocol. The aPHQ-9, the first culturally adapted, nationally validated, freely available depression screening tool for use by Indigenous people, appears to be acceptable and feasible to use.

**Trial registration:**

Australian New Zealand Clinical Trial Registry ANZCTR12614000705684, 03/07/2014.

## Background

In Australia, an estimated 6.2% of the population have experienced depression or another affective disorder during the previous 12 months [1], and Aboriginal and Torres Strait Islander people (hereafter referred to as Indigenous) are nearly three times as likely to experience high/very high levels of psychological distress than non-indigenous Australians [2]. The national prevalence rates and burden of depression among Indigenous communities remains unclear, in part because the K10 [3] used to capture data [2] measures psychological distress [4] and was developed using Western concepts of mental health that do not incorporate Indigenous definitions of social and emotional wellbeing (SEWB) [5]. Previous work has identified low rates of screening for depression and other SEWB problems (mean screening rate of 26.6%) in Indigenous-focused primary healthcare (PHC) services [6], limiting opportunities to identify and treat depression. There are several studies [7–10] where researchers have adapted and validated culturally-appropriate tools for detecting depression among Indigenous peoples. To the best of our knowledge these studies have not included process evaluations or formally captured staff and patients’ perspectives of the adapted and validated tools. Furthermore, the tools have not been validated outside of the Indigenous communities in which they were adapted.

We designed Getting it Right: the validation study [11] (hereafter Getting it Right) to determine the validity of the culturally-adapted 9-item Patient Health Questionnaire (aPHQ-9) [12, 13] as a depression screening tool for use by Indigenous people. Getting it Right was conducted in 10 Indigenous-focused PHC services (hereafter participating services) nationally between 2014 and 2016. Results from Getting it Right indicated that when used with a cut point of 10 (as per the original PHQ-9 algorithm) the aPHQ-9 has a sensitivity of 84% (95% CI 74 to 91%) and specificity of 77% (95% CI 71 to 83%) [14].

Process evaluations [15] are increasingly being conducted alongside research projects [16, 17] to systematically identify whether study protocols are followed as expected as well as other factors such as the acceptability of the intervention under investigation. Process evaluations can also identify important contextual factors surrounding research when and if any unintended consequences arose as a direct result of the research, such as additional burden on staff or insufficient resourcing, which may impact on the research not being conducted according to the study protocol. In addition, exploring and documenting research using process evaluations in this way provides opportunities to maximise lessons learned from one project to the next.

Various challenges for conducting SEWB research with Indigenous communities have been reported including high staff turnover [18] difficulties hiring staff to work on reserach [19] and changes in the service's priorities over time [20]. A previous study which aimed to validate an adapted depression screening tool for use by Aboriginal people highlights the impact of these challenges on their research [7]. Study recruitment was stopped early after 34 participants due to time and resource limitations. Consequently, exploring staff and patients’ perspectives about research via a process evaluation may be useful when planning and conducting future research. In this paper we present the results of a process evaluation to determine if Getting it Right was conducted as intended, and PHC staff and research participants’ perspectives about using the aPHQ-9 at the 10 participating services.

### Aim

To explore PHC staff perceptions about the conduct of Getting it Right, and staff and research participants’ perspectives of the perceived acceptability and feasibility of the aPHQ-9.

## Methods

The methods of Getting it Right [11] and the associated process evaluation [21] have been described previously in published protocols.

### Participant selection

Patients were recruited to Getting it Right by staff nominated by participating services and completed one interview using the aPHQ-9 [12] and a second interview using the semi-structured MINI International Neuropsychiatric Interview 6.0.0 (MINI) [22] between May 2015 and November 2016. The results of the two interviews were compared to determine the validity of the aPHQ-9 screening tool results against the criterion standard (the MINI). It was established that the aPHQ-9 was a valid screening tool for depression with Aboriginal and Torres Strait Islander peoples [14].

Staff were recruited to the process evaluation after Getting it Right recruitment was completed (before the validation results were available) and once approvals from participating services and ethics committees were received, between November 2016 and June 2017. The staff member coordinating Getting it Right at each respective participating service purposively identified [15] staff involved with Getting it Right (as interviewers or managers of interviewers) at their service as potentially eligible to take part. In addition, community members from one community group who reviewed and approved the research at their service were also invited to take part in a group interview. The coordinating staff member invited staff and Indigenous community members to complete qualitative semi-structured grounded theory [23] interviews with SF. If staff and community members were willing to take part, then informed consent was completed, and an interview time was scheduled.

### Data collection

Participant feedback was collected immediately after the aPHQ-9 interview using pre-specified questions about perceptions of the aPHQ-9 on the structured case report forms. The pre-specified questions asked directly about the number of questions, and if questions were easy to answer, easy to understand, the response categories made sense, they had time to answer the questions, and they felt comfortable answering the questions. Participants were then given the opportunity to provide free-text feedback about any part of Getting it Right. In addition to the aPHQ-9 [12] and the feedback questions, our case report forms included the (unmodified) 10th PHQ-9 question *‘If you checked off any problems, how difficult have these problems made it for you to do your work, take care of things at home, or get along with other people?.’* We also asked seven additional questions identified during the original adaption study [13] as key features of depression by Indigenous Australian people. These questions asked about anger, weakened spirit, homesickness, irritability, excessive worry, rumination, and drug or alcohol use.

Staff interviews were conducted in a confidential setting, in-person, at participating services or via the telephone, using an interview guide. SF and AME piloted the first interview guide (available on request). Staff interviews included questions about staff experiences participating in Getting it Right; the training delivered to conduct the research; if they perceived that deviations from the protocol occurred; the impact of the research on their workload; and their perceptions of screening, recruitment and using the aPHQ-9 during research interviews. The community group interview included questions about their perceptions of reviewing and approving for Getting it Right to be conducted in their community, participating in Getting it Right and of the aPHQ-9. Staff and community interviews were recorded and transcribed verbatim.

### Data analysis

Participant feedback to the pre-specified questions were analysed to identify the frequency that each statement was selected by participants. Free-text participant feedback was coded and themes were inductively developed. Using grounded theory approaches [23] data from staff and community member interviews were open coded by SF as soon as possible after each interview or group of interviews and themes were inductively developed. Independent double coding of 10 (25%) interviews was completed by two co-authors (GG and AME). A record of codes, their properties, interpretation, and author feedback were kept as memos, grouped into themes and integrated into subsequent interview guides (three interview guides were developed). In line with grounded theory approaches, codes, themes and memos were constantly compared throughout the process evaluation. Process evaluation interviews continued until all potential staff or community members who wished to, took part, rather than after concept saturation, to give all potential participants the opportunity to contribute. Staff interview data were triangulated with participant feedback and field notes taken by SF. NVivo 10 for Windows software [24] was used to manage data.

### Ethics and oversight of the process evaluation

An Advisory Group of Indigenous researchers and staff was convened to provide cultural oversight and local input into this process evaluation. The Advisory Group was provided with reports of the interviews and their feedback on reports was kept in memos and integrated into study findings. SF is a female registered nurse and was a PhD candidate who had completed training in qualitative data collection, analysis and reporting. Staff and community members had worked with SF in her role as the Getting it Right project manager for between one and three years. MH, GG and AB were investigators on Getting it Right.

Ethical approval details are available in the published study protocol [21]. Each participating service provided consent to participate in this process evaluation, participants provided informed consent and this process evaluation was conceived, designed and conducted while following the Values and Ethics Guideline [25].

In this paper ‘patient’ is defined as an individual using a PHC service in general or before consenting to participate in Getting it Right, and ‘participant’ as any patient who provided informed consent to participate in Getting it Right. Staff and community members who were interviewed are referred to as staff or community members. The term ‘Indigenous peoples’ is respectfully used in this paper and refers to all Aboriginal and/or Torres Strait Islander Peoples of Australia. We acknowledge the cultural diversity of Australia’s Indigenous First Peoples, and they do not represent a homogenous group.

## Results

Process evaluation interviews were completed with 36 staff (34 as individual interviews and two at the same time as a group interview) including: managers (*n* = 10), Aboriginal Health Workers (AHW *n* = 9), Allied Health Staff (*n* = 8), Research Coordinators (*n* = 5), and General Practitioners (GPs *n* = 4) from nine of the 10 participating services resulting in 1324 min of transcribed interviews. Four community members from one participating service completed a group interview. The staff members coordinating the research did not report any staff who did not agree to participate, however four of the eight community members who originally agreed to attend the group interview did not arrive and reported they were too busy or unavailable. Substantial staff turnover and organisational change occurred at the tenth participating service after Getting it Right was completed, so staff chose not to participate in the process evaluation while the new managers modified their processes to manage research. Staff and community member demographic information is presented in Table 1. No new open codes were identified in the final two interviews from one service, indicating data saturation.

**Table 1:** Demographic information for staff and community members who completed qualitative interviews

**Staff characteristics**	*N* = 36
Gender	
Female	24
Ethnicity	
Indigenous	17
Years working at participating health service	
Less than one year	0
1 to < 2 years	11
2 to < 3 years	2
3 to < 5 years	6
≥ 5 years	13
Data unavailable	4
**Community members’ characteristics**	*N* = 4
Gender	
Female	2
Ethnicity	
Indigenous	4

Feedback was collected from 500 participants who consented to participate in Getting it Right and completed both research interviews. Four hundred and ninety three participants responded to all six of the pre-specified‘perception of the aPHQ-9’ feedback questions and seven participants did not respond to one question each (99% completion rate). Approximately 20% of participants provided free-text feedback, which mostly related to the seven additional questions and the unmodified 10th ‘difficulties question’.

### PHC staff perceptions about the conduct of Getting it Right according to the study protocol

Most staff reported that they conducted the research according to the study protocol and that recruitment processes, data entry and safety follow-up processes aligned with the study protocol. The exceptions were staff from two services who reported sometimes recruiting opportunistically rather than recruiting consecutive patients attending the service as outlined in the main study protocol. Some staff reported speaking to patients who they knew from previous interactions, believing their existing relationship meant that these patients were more likely to participate:

I just talked to people I already had a relationship with … I found it was easier to recruit people who knew me and trusted me already rather than when I tried to recruit people in the clinic who I hadn’t met before, not many of them were agreeable. (Nurse, non-indigenous, site E)

According to the manager at this service, recruiting people whom they already knew led to honest conversations, which resulted in accurate research data. The manager reported that this approach was necessary to overcome the hurdles they experienced in reaching their recruitment target of 50 participants at their service:

Everyones' [staff] on holidays and you’ve got four clients that need INRs [blood test] and you’re trying to validate [recruit participants] … sometimes research doesn’t take the priority … We just have to be able to be opportunistic. (Manager, Indigenous, site E)

A review of the Getting it Right participant data showed a normal spread in demographics and illness burden across participants and services (data available on request).

Staff also reported that patients’ medical and personal histories influenced how patients responded when introduced to the research. Some patients who were unwell were unwilling to participate and patients with complex medical histories were occasionally approached about the research multiple times because of their frequent attendence at the service. Staff perceived that patients’ personal histories influenced their likelihood to participate because:

People who were reluctant were suspicious that answering [the interview questions] was going to affect their lives, that the government would come and check them out because of their answers. (Indigenous, female, AHW, site F)

Analysis of the screening logs showed a participation rate of 59% with reasons for non-participation including: i) declined, no reason documented, (64%); ii) ineligible, (33%); and iii) reason unclear, (3%).

### PHC staff and participants’ views about the acceptability and feasibility of the aPHQ-9

Over half the interviewed staff reported that they would use the aPHQ-9 when speaking with patients about their SEWB in the future, if it was found to be valid. Many reported that participants responded well to the aPHQ-9 because it used simple or clear language:

They [participants] thought that the aPHQ-9 was better – the ones that mentioned it – and I didn’t ask them mostly; they would offer that information. They did say “it is a lot easier to understand.” (Aboriginal Health Worker, male, Indigenous, site D)

Participants reported they were comfortable with the number of questions asked (90%), the questions were easy to understand (87%), easy to answer (82%), the response categories made sense (89%), they felt comfortable answering the questions (91%), had time to answer the questions (98%) and the questions were not too personal (86%). Five percent of respondents thought the questions were too personal and that they didn’t really want to answer them, 8% thought some questions were too personal and 1% didn’t care. However, 497 participants answered all aPHQ-9 questions with the remaining three participants missing one question each [14]. In the free-text feedback six participants reported that they expected the questions to be more personal.

Four staff and one participant reported that the term ‘spirit’ was not used in their community or that it was not relevant when used in the following aPHQ-9 question: ‘*have you been feeling unhappy, depressed, really no good, that your spirit was sad?’* and the additional question: *‘Have you felt that your spirit was weak?’* Conversely, one participant reported they:

Love [d] the way is asked with the word SPIRIT. (Indigenous participant, male, 55 years)

Eleven participants recommended adding questions to the aPHQ-9. Recommendations included questions about mobility or physical health problems, living situations, relationships, socialisation and isolation, culture, employment, education, current treatment plans for anxiety or depression, or questions to identify participants’ biggest worry, difficulty getting up in the morning, or perceptions of having a good and bad day. Five recommended including a comment box for additional information that some participants may wish to provide. Two participants recommended including more response options (currently ‘none, a little bit, most of the time, all of the time’). Another two recommended limiting options to yes/no. Some staff reported that the current response options were appropriate, while others suggested limiting the options to yes/no because they perceived that some participants found it challenging to select a response from the four options:

Because [the multiple options] gives them the option of saying, ‘well, look, sometimes … ’ (Manager, Indigenous, site E)

## Discussion

This reserach has shown that the Getting it Right study, which found the aPHQ-9 screening tool for depression was valid for use for and by Indigenous peoples [14], was conducted predominantly as outlined in the study protocol and that the aPHQ-9 was well accepted by the PHC staff and participants, and is considered acceptable and feasible to use. The non-consecutive recruitment that occurred sometimes at two services did not appear to result in biased samples at either service.

In response to our question ‘were the questions too personal?’ 25 participants (5%) selected the response ‘yes the questions were all too personal and I didn’t really want to answer them’. Despite this, in the free-text response space, others indicated that they expected the questions would be more personal than they were. This indicates that overall the aPHQ-9 was generally accepted and participants were willing to speak about wellbeing during the study.

Feedback from some staff and participants about the term ‘spirit’ was mixed. In the aPHQ-9 development work completed in central Australia [12, 13] and subsequent qualitative work exploring the experiences of men with depression [26], spirit was identified as the most appropriate term to encapsulate the emotional expression of depressed mood experienced by Aboriginal men. Our results suggest that the word spirit may be understood or used differently among heterogeneous Indigenous communities across multiple geographical regions. The word spirit is used in the second question of the aPHQ-9 alongside three other terms (unhappy, depressed, really no good) to enhance understanding of the same concept, so even in communities where ‘spirit’ may not be used, local alternatives might be available that could replace this word.

These results which show that the aPHQ-9 was accepted by most staff and participants involved with Getting it Right indicate that the aPHQ-9 may provide PHC services with a culturally valid resource to use which may increase the low rates of screening for depression in PHC [6]. Efforts to increase screening for depression should be supported by:

- Pathways of care for those requiring further assessment of SEWB [27],
- Access to appropriately skilled clinicians who can conduct psychiatric assessments to determine whether a diagnosis and plan treatment are warranted [28],
- Training for clinicians to prepare them for managing discussions about depression and other mood disorders [27],
- Allocation of adequate staff time to complete discussions around SEWB.

This reserach has demonstrated that conducting multi-site Indigenous-focused SEWB research is feasible and staff and patients were willing to participate in Getting it Right. Although existing relationships between staff and participants may have led to some staff recruiting opportunistically rather than consecutively, these relationships may have increased the likelihood of patients taking part, and of having honest conversations. This is consistent with findings from a qualitative study involving staff at two Aboriginal Community Controlled Health Services in which staff reported that Indigenous community members engaged more with research when they knew and trusted research staff, and that this led to greater and more accurate data collection [29]. The high rates of high/very high levels of psychological distress in Indigenous communities [2] emphasises the need for Indigenous-focused SEWB research to be planned and conducted by or in close collaboration with locally-based staff who understand the ‘lay of the land’ [30] as this may enhance participation, and is also crucial for research to be ethical [25]. The insights into staff and participant perspectives gained from this process evaluation demonstrates the value of conducting process evaluations alongside research. A similar approach may be useful to other researchers as it provides opportunities to explore and uncover the context surrounding research projects, how research protocols are implemented and may assist with maximising learnings from one project to the next.

A strength of the process evaluation is the in-depth knowledge of Getting it Right and its processes by the authors who were PHC service staff at participating services (JF, BH, MS and KD), investigators (MH, GG, NG and AB) and the project manager (SF). However, we also acknowledge that our varying roles could produce different biases, such as potentially influencing staff to provide positive responses about the study conduct, their experiences using the aPHQ-9 and how useful they found the measure. With this in mind, particular focus was given to probing for themes that indicated potential problems with the conduct of the study and/or using the aPHQ-9. An alternative approach could have been for a person independent of Getting it Right to conduct the process evaluation. This would introduce different challenges relating to a lack of knowledge of the study, and extended timeframes to develop relationships with PHC service staff sufficient for them to agree to participate in in-depth interviews about their research practices.

Further information on patient perspectives of the aPHQ-9 [12] may be gained from speaking directly with participants. However, interviews would need to be conducted immediately after participants completed the aPHQ-9 [12], and before the MINI [22]. This would have added significantly to participant burden and potentially contaminated responses to the MINI interview. In addition, the large number of patients and health services involved in Getting it Right meant it was beyond the scope of this process evaluation.

## Conclusions

As the first culturally adapted, nationally validated, freely available depression screening tool for depression for use by Indigenous people, the aPHQ-9 may be useful in PHC to identify those who need further assessment of their mood, or to work through individual questions to facilitate conversations with people about their mood. However, further research is needed to explore its consistency over time (test re-test reliability) and its performance when monitoring depression outcomes (response to treatment).

## Data Availability

The datasets generated and/or analysed during the current study are not publicly available due to the sensitive nature of the research.

## Abbreviations

AHW: Aboriginal Health Workers
aPHQ-9: adapted Patient Health Questionnaire-9
GP: General Practitioners
MINI: MINI International Neuropsychiatric Interview 6.0.0
PHC: Primary healthcare
SEWB: Social and emotional wellbeing
